# Hospitalization Rates and Post-Operative Mortality for Abdominal Aortic Aneurysm in Italy over the Period 2000–2011

**DOI:** 10.1371/journal.pone.0083855

**Published:** 2013-12-30

**Authors:** Luigi Sensi, Dario Tedesco, Stefano Mimmi, Paola Rucci, Emilio Pisano, Luciano Pedrini, Kathryn M. McDonald, Maria Pia Fantini, on behalf of the AAA-Working Group

**Affiliations:** 1 Department of Surgery, Vascular Surgery Unit, Maggiore Hospital, Bologna, Italy; 2 Department of Biomedical and Neuromotor Sciences, Alma Mater Studiorum - University of Bologna, Bologna, Italy; 3 Stanford Center for Health Policy/Center for Primary Care and Outcomes Research, Stanford University, Stanford, California, United States of America; 4 Vascular Surgery Unit, Maggiore Hospital, Bologna, Italy; San Raffaele Scientific Institute, Italy

## Abstract

**Background:**

Recent studies have reported declines in incidence, prevalence and mortality for abdominal aortic aneurysms (AAAs) in various countries, but evidence from Mediterranean countries is lacking. The aim of this study is to examine the trend of hospitalization and post-operative mortality rates for AAAs in Italy during the period 2000–2011, taking into account the introduction of endovascular aneurysm repair (EVAR) in 1990s.

**Methods:**

This retrospective cohort study was carried out in Emilia-Romagna, an Italian region with 4.5 million inhabitants. A total of 19,673 patients hospitalized for AAAs between 2000 and 2011, were identified from the hospital discharge records (HDR) database. Hospitalization rates, percentage of OSR and EVAR and 30-day mortality rates were calculated for unruptured (uAAAs) and ruptured AAAs (rAAAs).

**Results:**

Adjusted hospitalization rates decreased on average by 2.9% per year for uAAAs and 3.2% for rAAAs (p<0.001). The temporal trend of 30-day mortality rates remained stable for both groups. The percentage of EVAR for uAAAs increased significantly from 2006 to 2011 (42.7 versus 60.9% respectively, mean change of 3.9% per year, p<0.001). No significant difference in mortality was found between OSR and EVAR for uAAAs and rAAAs.

**Conclusions:**

The incidence and trend of hospitalization rates for rAAAs and uAAAs decreased significantly in the last decade, while 30-day mortality rates in operated patients remained stable. OSR continued to be the most common surgery in rAAAs, although the gap between OSR and EVAR recently declined. The EVAR technique became the preferred surgery for uAAAs since 2008.

## Introduction

Abdominal aortic aneurysm (AAA) is a common, progressive and potentially fatal disease with partly unknown aetiology [1]. If left untreated, the aortic wall weakens and becomes unable to withstand the forces of the luminal blood pressure, resulting in progressive dilatation and rupture [2].

During the 20th century, hospitalization and mortality rates from AAAs increased in Western countries [3]–[5]. Although advances in diagnostic techniques, such as ultrasonography, may have contributed to this trend, the rise of mortality and morbidity from AAAs could reflect a true increase of incidence, likely due also to increasing risk factors, such as smoking and age [6]–[8]. Recently, studies from England and Wales, Australia, New Zealand and Sweden have reported declines in AAAs incidence, prevalence and mortality during the last decade [9]–[12].

The detection of AAAs may be incidental, may result from a screening program (unruptured AAAs, uAAAs) or occur dramatically through the rupture of the aortic wall (ruptured AAAs, rAAAs). Ruptured aneurysm is associated with a very high mortality rate (about 4 deaths per 100,000 in England and Wales in the years 2001–2009) [9].

Until the 1990s, aneurysms were treated by open surgical repair (OSR). The introduction in1991 of endovascular aneurysm repair (EVAR) [13] has led to changes in access to surgery and in quality of care and economic costs [14], [15]. As EVAR technology has improved, the number of EVAR has grown so as to exceed, in the American context [16], the number of OSR. In Europe this transition has not yet occurred, but the trend seems headed in that direction [17]. In the last decade several studies have investigated postoperative mortality related to EVAR and all have roughly confirmed that EVAR achieves better short-term outcomes than OSR, but does not show significantly better results in terms of long-term outcomes and costs [18], [19].

Up to now, these topics have been inadequately investigated in Mediterranean countries. This study aims to provide the hospitalization and mortality rates for AAAs in the Northern Italian Emilia-Romagna region with about 4.5 million inhabitants for the years 2000–2011, using administrative data sources which are increasingly used to compare hospital performance and to examine patient outcomes in Western countries [20].

## Materials and Methods

### Ethics Statement

The study was carried out in conformity with the regulations on data management of the Regional Health Authority of Emilia-Romagna, and with the Italian law on privacy (Art. 20–21, DL 196/2003) (http://www.garanteprivacy.it/web/guest/home/docweb/-/docweb-display/docweb/1115480, published in the Official Journal no. 190 of August 14, 2004) which explicitly exempts the need of ethical approval for anonymous data (Preamble #8).

Data were anonymized prior to the analysis at the regional statistical office, where each patient was assigned a unique identifier. This identifier does not link to the patient’s identity and other sensitive data. As anonymized administrative data are used routinely for health-care management no specific written informed consent was needed to use patient information.

The data set will be made freely available upon request.

### Population, Data and Outcomes

This retrospective cohort study used the hospital discharge records (HDR) database of Emilia-Romagna region, where information on gender, birth date, admission and discharge date, status at discharge, diagnoses and procedures is recorded for all patients discharged. Patients discharged between January 1^st^, 2000 and December 31^st^, 2011 with primary diagnosis of abdominal aortic aneurysms were selected for the analyses. Hospital discharge records of patients under 18 years or not resident in Emilia Romagna region were excluded. The ICD-9-CM diagnostic codes used for the present study were 441.3 (abdominal aneurysm, ruptured), 441.4 (abdominal aneurysm without mention of rupture), 441.5 (aortic aneurysm of unspecified site, ruptured) and 441.9 (aortic aneurysm of unspecified site without mention of rupture).

AAAs were classified into uAAAs and rAAAs using the following criteria: uAAAs were patients with a primary diagnosis 441.4 or 441.9 (excluding patients with secondary diagnostic codes 441.3 or 441.5); rAAAs were patients with a primary diagnosis 441.3 or 441.5 (excluding patients with secondary diagnostic codes 441.4 or 441.9).

Thirty-day in-hospital mortality was estimated in patients with AAAs who received a procedure coded as 38.34 (resection of vessel with anastomosis, aorta), 38.44 (resection of vessel with replacement, aorta ), 38.64 (other excision of vessels, aorta, abdominal ), 39.52 (other repair of aneurysm), 39.71 (endovascular implantation of graft in abdominal aorta), or 39.79 (other endovascular procedures on other vessels). This outcome was defined as a death occurring for any cause within 30 days from surgery during the index hospitalization or any subsequent hospitalization.

Information on comorbidity in the 2 years before the index hospitalization and at admission was obtained through data linkage of hospital discharge records for each patient.

From 1998 to 2004 Italian health authorities adopted the ICD-9-CM, 1997 version, which did not include the EVAR procedure codes. Since 2005, the ICD-9-CM, 2002 version was adopted, in which the EVAR procedure codes were included. Thus it was possible to distinguish EVAR (ICD-9-CM codes 39.71 or 39.79) and OSR (ICD-9-CM codes 38.34 or 38.44 or 38.64 or 39.52) procedures starting from 2006.

### Validity of the Algorithm for Case Definition

The sensitivity of the algorithm for case identification was examined by linking HDR with the clinical database of the Vascular Surgery Unit of Maggiore Hospital, including 523 patients who underwent surgery between 2000 and 2005. We found that the algorithm captured 69% of cases. Further examination of the diagnostic/procedure codes revealed that 14 ruptured aneurysms had been coded as 441.02 (dissection of abdominal aorta), and for 78 cases the procedure 39.52 (other repair of aneurysm) had been used. Therefore we modified the algorithm to include diagnosis 441.02 and procedure 39.52. Broadening the inclusion criteria increased the sensitivity of the algorithm to 88%. Therefore, although the HDR database covers 100% of hospital discharges in the region, the algorithm based on a primary diagnosis of AAA and some specific procedures does not identify all operated AAA cases.

However, studies using administrative data in Emilia-Romagna region show that HDRs are reliable [21]. To assess the accuracy of 30-day in-hospital mortality data as a proxy of 30-day mortality, HDR were linked to the regional mortality registry for the period 2000–2009. Only 1% of deaths within 30 days occurred outside of the hospital. Therefore 30-day in-hospital mortality may be considered as an accurate proxy of 30-day mortality when death registry data are unavailable to researchers (e.g., due to time lag of two or more years).

### Statistical Analysis

Crude hospitalization rates were calculated for each year as the number of patients hospitalized divided by the number of resident adults. The hospitalization rates adjusted for age and gender were calculated using as reference population the resident adults on January 1^st^, 2006.

The 30-day mortality rates for uAAAs and rAAAs were estimated using a risk-adjusted Poisson multivariate regression model including as covariates sex, age, and comorbidity in the index admission and in the admissions of the previous two years. The trend of mortality and hospitalization rates in the study period was estimated using linear regression.

The study period was limited to the years 2006–2011 for 30-day mortality rates for OSR and EVAR procedure in uAAAs when EVAR procedure coding was reliably available. Relative risks were used to compare the 30-day mortality rates for OSR and EVAR procedures. Statistical analysis was carried out using SAS, version 8.2 (SAS Institute Inc., USA).

## Results

### Baseline Characteristics

The analysis included 19,673 patients with AAAs, 16,903 of whom (85.9%) were uAAAs and 2,770 (14.1%) rAAAs. Patients underwent AAA repair in 22 hospitals, 14 of which had a vascular surgery department.

### Hospitalization of Patients with AAAs

**Figure 1:**
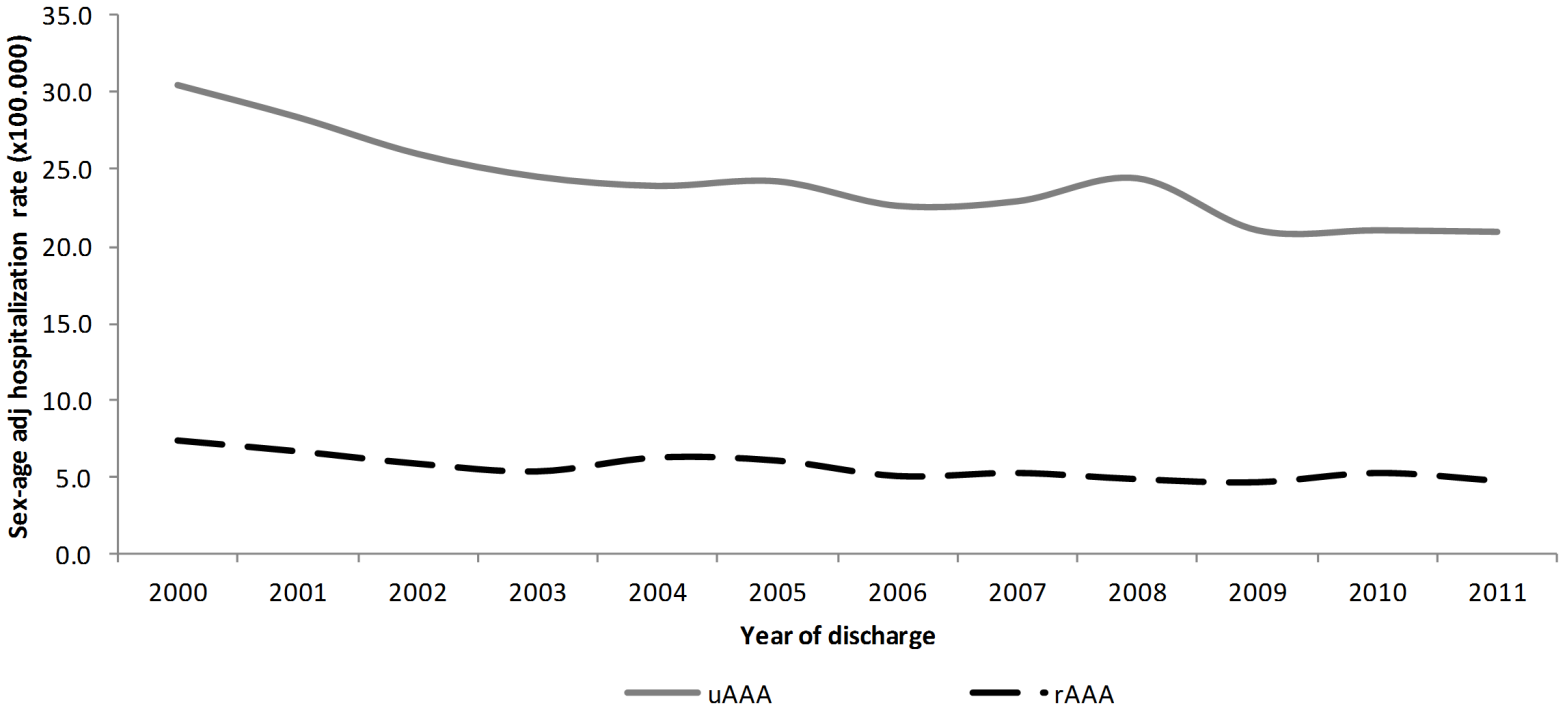
Age- and gender-adjusted hospitalization rates for uAAAs and rAAAs. uAAAs, unruptured abdominal aortic aneurysm; rAAAs, ruptured abdominal aortic aneurysm.

Age- and gender-adjusted hospitalization rates for uAAAs in Emilia-Romagna region decreased significantly from 30.5 in 2000 to 20.9 per 100,000 in 2011, on average by 2.9% per year (p<0.001). Similarly, adjusted hospitalization rates for rAAAs decreased significantly from 7.4 in 2000 to 4.8 per 100,000 in 2011, on average by 3.2% per year (p<0.001) (Figure 1).

### Percentage of Patients Aged 75 Years and Over

**Figure 2:**
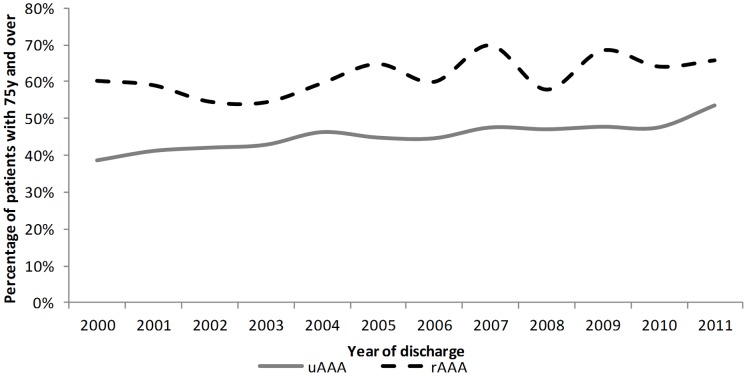
Percentage of patients 75 years and over.

For both uAAAs and rAAAs, we found an increase in the percentage of patients aged ≥75 years during the period 2000 to 2011. This percentage increased by 1.0% per year (p<0.001) for uAAAs and 0.9% per year (p = 0.032) for rAAAs (Figure 2).

### Thirty-day Mortality of Operated AAAs Patients

The number and characteristics of operated patients are reported in Table 1. Both crude and adjusted 30-day in-hospital mortality rates for uAAAs and rAAAs showed non-significant fluctuations over the study period (Table 2). The mean adjusted 30-day mortality rate for uAAAs was 1.87% (range 1.21–2.74%) and the rAAAs adjusted 30-day in-hospital mortality rate was 36.35% (range 27.46–43.00%).

**Table 1:** Characteristics of patients undergoing AAA repair.

	uAAAs	rAAAs
Patients	7,869	1,497
Male n (%)	7,198 (91.5%)	1,260 (84.2%)
Age (±SD)	72.5 (±7.68)	76.3 (±9.26)
Hypertension at index discharge	3,912 (49.7%)	420 (28.1%)
Heart failure at index discharge	67 (0,8%)	49 (3.3%)
Cardiac arrhythmia at index discharge	622 (7.9%)	187 (12.5%)
Hematological diseases at index discharge	402 (5.1%)	232 (15.5%)
COPD at index discharge	2,181 (27.7%)	233 (15.6%)
Previous chronic kidney disease	401 (5.1%)	105 (7.0%)
Previous COPD	923 (11.7%)	126 (8.4%)
30-day mortality after AAAs repair	148 (1.9%)	543 (36.3%)

M, males; COPD, chronic obstructive pulmonary disease.

**Table 2:** Thirty-day in-hospital mortality rates.

	uAAAs		rAAAs	
Year of discharge	Crude 30-daymortality rate (%)	Adjusted^a^ 30-daymortality rate (%)	Crude 30-daymortality rate (%)	Adjusted^b^ 30-daymortality rate (%)
2000	1.99	1.84	35.71	34.83
2001	1.53	1.30	33.59	33.82
2002	2.42	2.02	36.92	37.67
2003	1.75	1.57	37.17	39.54
2004	2.45	2.13	37.06	35.73
2005	1.79	1.76	35.62	34.82
2006	2.48	2.74	29.91	27.46
2007	1.88	2.14	30.16	29.87
2008	1.59	1.81	35.29	38.01
2009	1.91	2.13	46.28	43.00
2010	1.60	1.88	37.30	38.74
2011	1.23	1.21	40.71	42.71
Trend (p-value)	−0.0459 (0.176)	0.0044 (0.904)	0.4129 (0.278)	0.4744 (0.239)

^a^ Adjusted for age, gender, OSR/EVAR, hypertension at index discharge, heart failure at index discharge, cardiac arrhythmia at index discharge, previous chronic kidney disease.

^b^ Adjusted for age, gender, OSR/EVAR, hypertension at index discharge, hematological diseases at index discharge, COPD at index discharge, previous COPD, previous chronic kidney disease.

OSR, open surgical repair; EVAR, endovascular aneurysm repair.

### Percentage of OSR/EVAR

These analyses were carried out in the subsample operated since 2006. Patients with an aorto uni-iliac endograft followed by a femoro-femoral crossover (N = 170, 1.8% recorded as both EVAR and OSR) were excluded.

**Figure 3:**
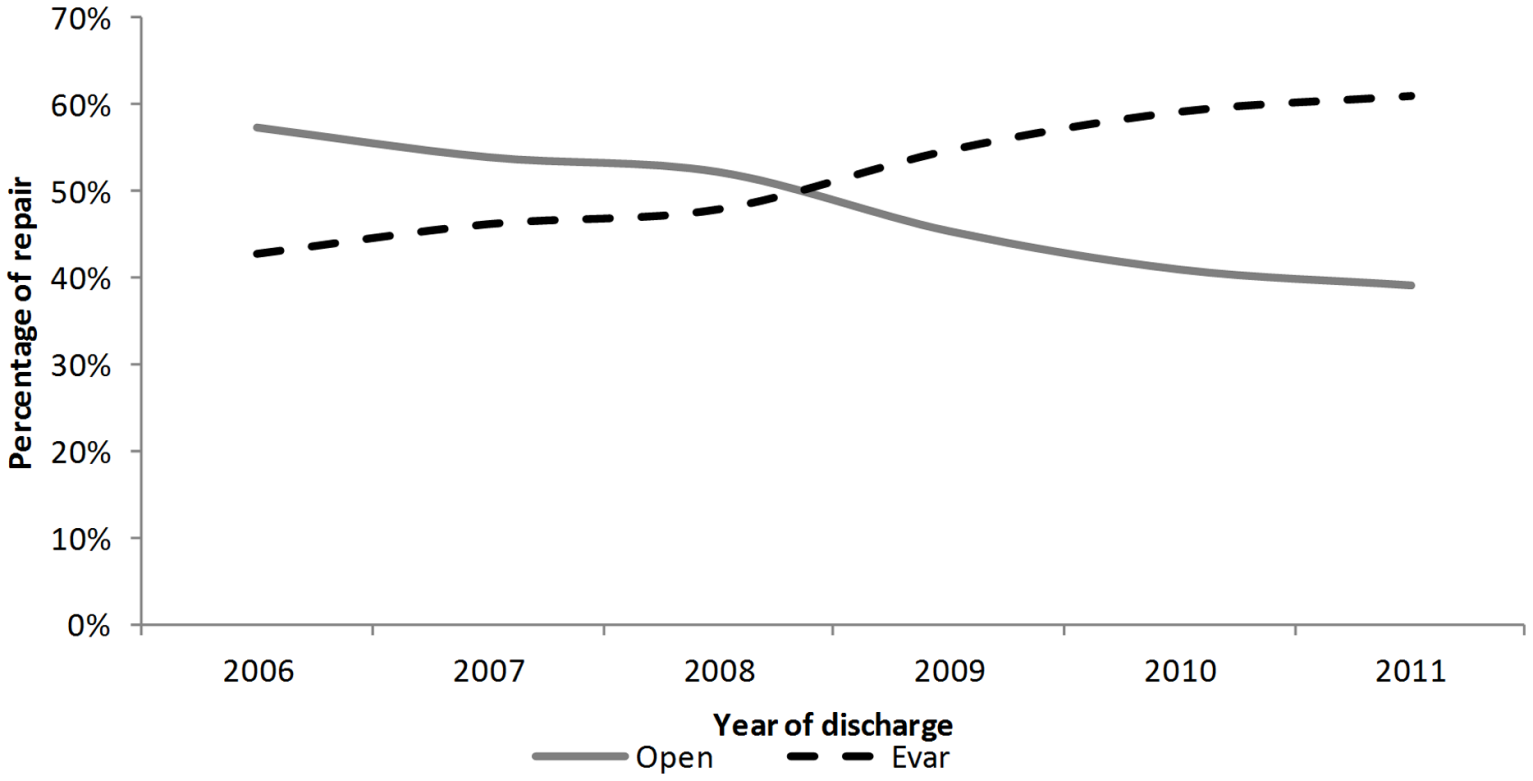
Percentage of OSR/EVAR in uAAAs.

The characteristics of patients undergoing each of procedure are provided in Table 3. The percentage of EVAR for uAAAs increased significantly from 42.7% in 2006 to 60.9% in 2011, with a mean change of 3.9% per year (p<0.001) (Figure 3). In this period, the mean length of hospital stay for uAAAs with OSR was significantly longer than the mean stay for uAAAs with EVAR (12.0 vs. 7.2 days, p-value <0.001).

**Table 3:** Characteristics of patients undergoing OSR and EVAR.

	uAAAs		rAAAs	
	OSR	EVAR	OSR	EVAR
Number of patients	1908	2063	513	182
Male n (%)	1737 (91%)	1885 (91.4%)	418 (81.5%)	150 (82.4%)
Age (±SD)	71.2 (7.41)	75.1 (7.39)	77.2 (9.44)	78 (8.94)
Hypertension at index discharge	987 (51.7%)	1220 (59.1%)	154 (30%)	62 (34.1%)
Heart failure at index discharge	21 (1.1%)	14 (0.7%)	20 (3.9%)	5 (2.7%)
Cardiac arrhythmia at index discharge	132 (6.9%)	218 (10.6%)	76 (14.8%)	28 (15.4%)
Hematological diseases at index discharge	149 (7.8%)	74 (3.6%)	72 (14%)	39 (21.4%)
COPD at index discharge	553 (29%)	609 (29.5%)	89 (17.3%)	39 (21.4%)
Previous chronic kidney disease	101 (5.3%)	154 (7.5%)	35 (6.8%)	16 (8.8%)
Previous COPD	182 (9.5%)	245 (11.9%)	37 (7.2%)	27 (14.8%)
30-day mortality after AAA repair	42 (2.2%)	28 (1.4%)	195 (38%)	61 (33.5%)

**Figure 4:**
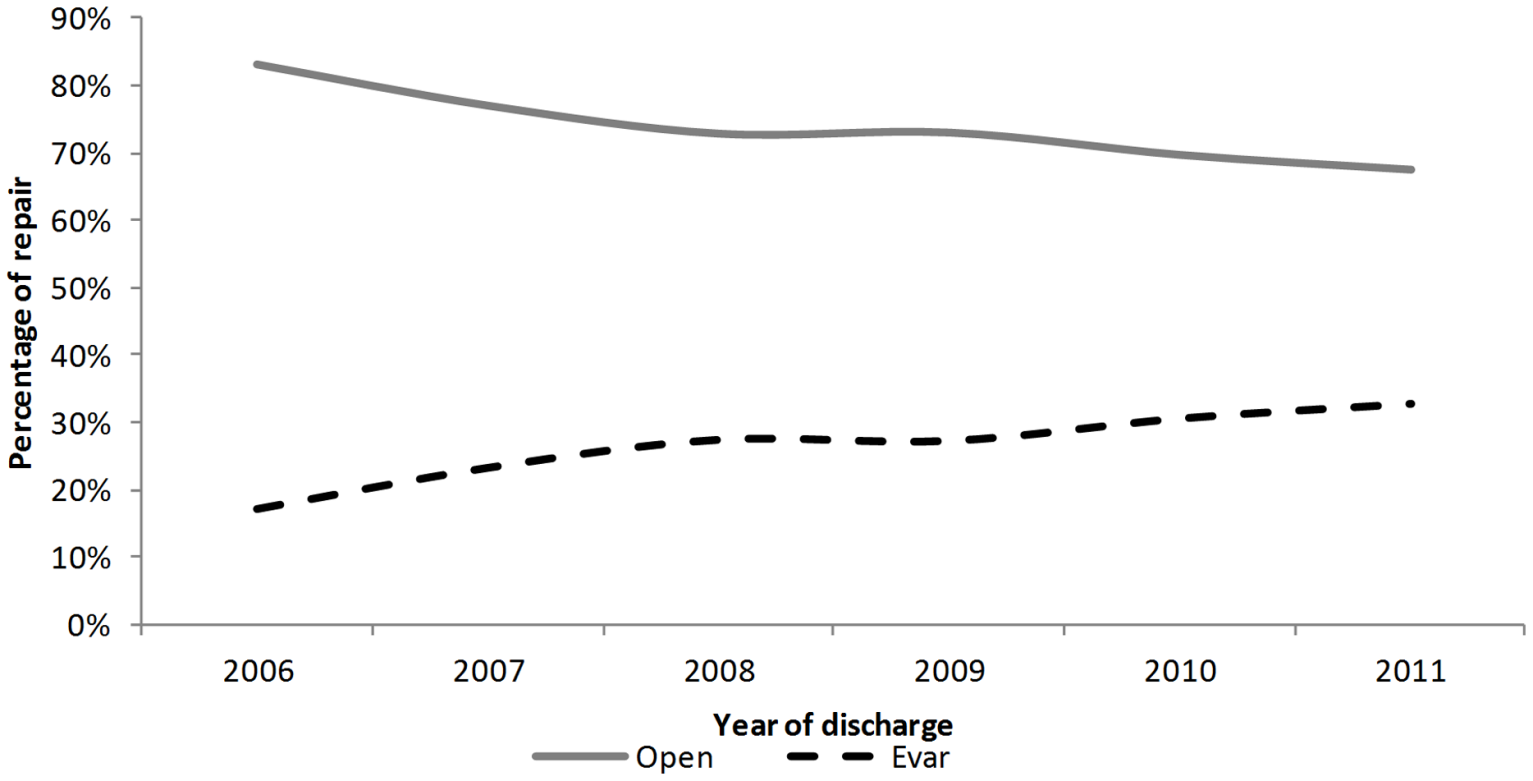
Percentage of OSR/EVAR in rAAAs.

The increase in the percentage of EVAR for rAAAs was smaller than for uAAAs, but still statistically significant, increasing from 17.1% in 2006 to 32.7% in 2011, with a mean change of 2.8% per annum (p = 0.003) (Figure 4). The mean length of stay for rAAAs with OSR was statistically longer than the mean stay for rAAAs with EVAR (16.5 vs. 11.6 days, p-value <0.001).

### Thirty-day Risk-adjusted Mortality of EVAR/OSR Repaired Patients

#### Unruptured AAAs

The difference in 30-day in-hospital mortality between OSR and EVAR of uAAAs was not significant, except for the year 2006 when a lower mortality was observed for EVAR. The 30-day in-hospital mortality adjusted rate after OSR decreased in the study period by a mean of 0.36% per year (though trend is not statistically significant: p = 0.087); the EVAR 30-day in-hospital mortality adjusted rate remained constant (Table 4).

**Table 4:** Thirty-day in-hospital mortality rate – uAAAs.

	OSR			EVAR			
year ofdischarge	Number ofoperated patients	Crude 30-daymortality rate (%)	Adjusted 30-daymortality rate (%)	Number ofoperated patients	Crude 30-daymortality rate (%)	Adjusted30-daymortality rate (%)	aRR (p-value)
2006	387	3.62	3.83	288	0.69	0.71	5.356 (0.013)
2007	331	1.81	1.92	283	2.12	1.91	1.006 (0.783)
2008	358	1.68	1.66	328	1.52	1.57	1.058 (0.874)
2009	288	2.43	2.30	347	1.44	1.49	1.545 (0.362)
2010	268	1.87	1.78	387	1.55	1.52	1.172 (0.757)
2011	276	1.45	1.26	430	0.93	1.01	1.251 (0.524)
Trend (p-value)		−0.2834 (0.146)	−0.3609 (0.087)		−0.0169 (0.907)	0.0071 (0.954)	

Adjusted for age, gender, OSR/EVAR, hypertension at index discharge, heart failure at index discharge, cardiac arrhythmia at index discharge, previous chronic kidney disease. aRR, adjusted risk ratio.

#### Ruptured AAAs

The 30-day in-hospital mortality of rAAAs did not differ between OSR and EVAR. While the 30-day mortality for the OSR increased from 2006 to 2011 by a mean of 2.93% (p = 0.024), that for EVAR did not change significantly (Table 5).

**Table 5:** Thirty-day in-hospital mortality rate – rAAAs.

	OSR			EVAR			
year ofdischarge	Number ofoperated patients	Crude 30-daymortality rate (%)	Adjusted 30-daymortality rate (%)	Number ofoperated patients	Crude 30-daymortality rate (%)	Adjusted 30-daymortality rate (%)	aRR (p-value)
2006	97	31.96	31.56	20	20.00	23.68	1.333 (0.287)
2007	96	33.33	33.59	29	20.69	20.73	1.621 (0.195)
2008	85	31.76	35.85	32	46.88	43.39	0.826 (0.129)
2009	83	49.40	44.58	31	38.71	41.26	1.080 (0.307)
2010	82	39.02	38.24	36	36.11	37.60	1.017 (0.764)
2011	70	45.71	47.57	34	32.35	29.53	1.611 (0.194)
Trend (p-value)		2.9560 (0.099)	2.9351 (0.024)		2.8526 (0.305)	2.2209 (0.383)	

Adjusted for age, gender, OSR/EVAR, hypertension at index discharge, hematological diseases at index discharge, COPD at index discharge, previous COPD, previous chronic kidney disease.

### Readmission Rates

Out of 2421 patients undergoing OSR, 269 (11.1%) were readmitted within 30 days of discharge (2.6% with a surgical DRG and 8.5% with a medical DRG). Of the 2245 patients undergoing EVAR, 277 (12.4%) were readmitted within 30 days of discharge (4.2% with a surgical DRG and 8.2% with a medical DRG).

## Discussion

This study evaluated the incidence and trend of AAAs, using HDR data. The results of the present study indicate a significant decrease in overall AAAs adjusted hospitalization rates both for uAAAs and rAAAs over the years 2000–2011. The temporal trend of 30-day mortality rates did not vary significantly for both patient groups.

Our hospitalization rates for uAAAs are consistent with those reported by Norman et al. [10] and Sandiford et al. [11], while other studies found steady or increasing trends [9], [17], [22]. The hospitalization rates for rAAAs in the present study are consistent with those for the English population in the last ten years [9].

AAAs may develop in a silent way and their detection may occur during a screening ultrasonography or incidentally; without screening programs, the hospitalization rate can be used as a proxy of incidence of AAAs, consistent with other studies [11]. While in many countries screening programs for AAAs have been implemented in the last 15 years [23], [24], in Italy implementation has been limited generally [25], [26]. However, in the Emilia-Romagna region, in the last decades, the use of ultrasonography has become widespread and diagnostic skills have improved, resulting in a greater ability to detect AAAs. This might have contributed to an increased diagnosis of incidental uAAAs in elderly patients. However, we observed a decrease in AAAs hospitalization rates. This decline might be related to reductions in risk factors given that smoking habits in Italy and in Emilia-Romagna region have steadily declined as a result of the enforcement by law of a comprehensive smoking ban in public places in 2005 [27]–[29]. The effect of this smoking ban on the reduction of acute cardiovascular events has been documented by Barone-Adesi et al. [30] and Cesaroni et al. [31]. In addition, during the study period a decreasing rate of lung cancer in Italy (starting in the 90′s) was observed, particularly among men (from 120/100,000 in 1990 to 80/100,000 in 2010) [32].

Unfortunately information on smoking habits is not available from HDR and could not be included in our analyses.

When we focused on elderly patients (aged 75 years or more), hospitalization rates increased over time both for uAAAs and for rAAAs. Regarding uAAAs, our results are consistent with other studies in which the authors explained this trend as a delayed clinical onset of illness, resulting from the combined effect of genetic factors and the decrease of smoking [33], [34].

Of note, in the present study adjusted mortality rates did not increase over the study period. However, because the number of elderly hospitalized patients increased over time, the steady mortality rates suggest good quality of care, particularly for this aged population.

It is possible that the decrease of hospitalization for uAAA is accounted for by several factors. In younger patients, some changes to care have been implemented during the study period, e.g. an increase in outpatient visits rather than hospital admission for diagnostic purposes, or the monitoring of small-diameter aneurysms rather than surgery (intervention is recommended only for AAA>5.5 cm) [35]. On the contrary, it is likely that the advent of EVAR has enabled safe aneurysm repair in elderly patients who might have been turned down for surgery before 2000 [22]. This could explain why we observed a decrease of overall hospitalizations (younger people effect), with a parallel increase of hospitalizations and interventions in elderly patients.

As to the choice of surgical procedure, OSR continued to be the most common surgery in rAAAs, although in the study period the gap between OSR and EVAR was reduced, even though some departments had limited hospital budgets for transitioning to EVAR. On the contrary, the EVAR technique became the preferred method for uAAAs since 2008, consistent with the literature [16]. The advantage of this surgical procedure is that it requires shorter hospitalizations (on average 5 days less), although it is more costly [15], [36]. However, the adjusted mortality did not decrease since the adoption of EVAR technique, in line with Gupta et al. [37] that used prospectively collected data from the National Surgical Quality Improvement Programme (for patients <60 years). On the contrary other studies reported a higher 30-day mortality for OSR [18], [38], [39]. Further studies are needed to assess the effectiveness and the cost-effectiveness of the two techniques, specifically in the Italian health care system.

Examination of readmission rates within 30 days of discharge showed that about one in ten patients were readmitted after OSR (11.1%) or EVAR (12.4%) repair. Of these readmissions, few had a surgical DRG (23% in OSR and 34% in EVAR), suggesting a good quality of surgical care. Our results are consistent with the findings of Greenblatt et al. [40], who reported 30-day readmission rates of 12.8% after EVAR and 13.3% after OSR. On the contrary, Casey et al. [41] found higher rates: 20.0% for OSR and 17.4% for EVAR. A recent U.S. study reports an almost 11% adverse event rate among OSR patients compared to less than 3% for EVAR patients [42].

Our results should be interpreted keeping in mind some important limitations. Statistical analysis of longitudinal observational data is challenging, complicated by the potential for unforeseen bias and confounding. Administrative databases have a limited ability to capture illness severity. This limitation is partially mitigated by our inclusion in the analyses of comorbidities in the index hospitalization and in the two previous years. However, in the present study, information on patients’ history of smoking, that has a strong impact on AAA, was not available. Second, the potential for inaccurate coding exists for any claims-based analysis. In order to increase the specificity of the selection algorithm, we included only patients with a primary diagnosis of AAAs. This did not allow to capture an estimated 12% with this diagnosis. Moreover we included among operated patients only those with one of the procedures selected for our algorithm. This led to exclude patients undergoing other procedures. Therefore, generalization of our results should be done with caution.

However, our data are population-based and are representative of the hospitalizations and procedures performed for this condition in the whole region.

## Conclusions

Similar to several previous studies in other countries, we find decreasing levels of AAAs in Italy, even in the setting of increased screening. Reductions in risk factors (e.g., less smoking) may be responsible for decreased disease burden.

Alongside reductions in disease burden at the population level, the health care delivery system is offering more patients surgical treatments. The Italian technology utilization trend is following that of other countries, though 30-day adjusted mortality rates are comparable for both procedural options, with rates for OSR lower than those reported elsewhere [18], [38], [39].

For future policy development as well as patient level decision-making, more comparative evidence on beneficial and adverse patient and economic effects of OSR, EVAR and no surgery is needed. Longitudinal data at the population level from regions such as Emilia Romagna could examine these outcomes over longer periods of time. Careful examination of conditions such as AAAs in different regions is likely to offer critical insight by exploring the complex interplay among patient, health delivery system and policy factors.
